# Importance of Antixenosis and Antibiosis Resistance to the Cabbage Whitefly (*Aleyrodes proletella*) in Brussels Sprout Cultivars

**DOI:** 10.3390/insects11010056

**Published:** 2020-01-17

**Authors:** Peter Hondelmann, Christina Paul, Monika Schreiner, Rainer Meyhöfer

**Affiliations:** 1 Institute of Horticultural Production Systems, Section Phytomedicine, Leibniz Universität Hannover, Herrenhäuser Straße 2, 30419 Hannover, Germany; Hondelmann@ipp.uni-hannover.de (P.H.);; 2 Department Plant Quality, Leibniz Institute of Vegetable and Ornamental Crops, Theodor-Echtermeyer-Weg 1, 14979 Grossbeeren, Germany; Schreiner@igzev.de

**Keywords:** plant resistance, Aleyrodidae, commercialised cultivars, integrated pest management

## Abstract

The cabbage whitefly *Aleyrodes proletella* (L.) (Hemiptera: Aleyrodidae) is an important pest of a wide range of vegetable Brassicas. Since the control of this pest is still challenging, new approaches such as the use of resistant cultivars are required. For this, we screened 16 commercialised Brussels sprout cultivars for resistance against this species. Antibiosis was tested with no-choice experiments in a climate chamber, using reproduction, mortality, longevity, developmental time and weight as parameters. Antixenosis was screened in three choice experiments with circular design in a greenhouse to detect cultivar preferences. A field trial with both antibiosis and antixenosis tests was done to verify results under natural conditions. Finally, for several cultivars, also the leaf glucosinolate concentrations were analysed. Cabbage whiteflies showed on certain cultivars significantly increased mortality, prolonged developmental times and reduced weights. Besides, some cultivars were significantly less infested. However, the incidence of antibiosis and antixenosis as well as the glucosinolate patterns were partly inconsistent. Although a number of moderately resistant cultivars could be identified, the detected resistance is certainly not strong and consistent enough as an exclusive measure of a plant protection strategy but might become a component of a multi-layered strategy against cabbage whiteflies.

## 1. Introduction

In the last decade, the cabbage whitefly *Aleyrodes proletella* (L.) (Hemiptera: Aleyrodidae) (CWF) developed to an economically important pest on several *Brassica* crops, such as Brussels sprout (*Brassica oleracea* L. var. *gemmifera* DC.), kale *(B. oleracea* L. var. *acephala* DC. f. *sabellica* L.), or Savoy cabbage (*B. oleracea* L. var. *sabauda* L.). Other brassicaceous crops are also affected, but this is usually of minor importance. The infestation causes substantial quality loss of the harvest due to large amounts of honeydew, exuvae and later growth of sooty moulds wax when pest population densities are high, but yield loss is also significant [1,2]. Although the reasons for the development of CWF to a major pest on cabbages are still uncertain, it is thought that milder winters and the huge increase of oilseed rape cultivation serving as a suitable winter host for this species play a decisive role [3,4,5,6]. However, also the biology of this species with 4–5 generations per year, the protected mode of life (beneath leaves) and the possibility to disperse over large distances [3,5] might have added to its success.

Nevertheless, reliable control strategies are lacking especially for organically grown *Brassica* crops due to the few registered and efficient pesticides [7]. Alternative control strategies mostly rely on nets with fine mesh size, which are expensive and can cause other problems, e.g., nets have to be temporarily removed for weeding, resulting in infestations with hemipteran and lepidopteran pests which are then protected against antagonistic insects. To overcome such problems, combinations with natural enemies (*Encarsia tricolor* Foerster (Hymenoptera: Aphelinidae), *Clitostethus arcuatus* Rossi (Coleoptera: Coccinellidae)) were also tested, but due to low reliability and high costs, it has not gained acceptance so far [8]. Conventional farmers, however, can still use efficient pesticides, such as spirotetramat or acetamiprid [9], but repeated applications and improved equipment (e.g., dropleg sprayers) are required for a sufficient control [7]. Finally, frequent pesticide applications contravene with today’s consumer concerns and political demands to reduce pesticide use and contamination of food and the environment with pesticide residues (e.g., [10,11]). Hence, new control approaches against cabbage whitefly are needed.

Host plant resistance can be a vital component of integrated pest management because of the relative ease of integration as part of an integrated pest management (IPM) strategy and the low impact on non-target organisms and the environment [12,13,14]. Plant resistance against cabbage whitefly recently moved into focus in the context of the increasing importance of this pest, but until now, strong resistance has been detected only in few landraces and wild cabbages, indicating potential for resistance breeding [15,16,17]. However, even in commercialised cultivars of different brassica crops, considerable differences in CWF resistance in terms of antibiosis (adverse effects of a plant on the survival, development, or reproduction of an arthropod) and antixenosis (non-preference reaction of an arthropod to a plant) according to [18] were found (e.g., [19,20]). Finally, in several studies, significant differences in the suitability of certain brassica host plants of CWF were found [21,22,23].

Among other resistance factors in Brassicaceae such as wax content or leaf hardness [12,24], glucosinolates are considered as a key factor of resistance, serving as chemical defence which can affect the performance and fitness of herbivores negatively and can also act as feeding deterrents [25]. However, little is known about the interactions of glucosinolates and CWF and its effects on plant attractiveness and CWF fitness.

In this study, we wanted to investigate if commercialised Brussels sprout cultivars have resistance traits against CWF and elucidate the role of glucosinolates in this context. Specifically, we asked the following questions:

1. Do cabbage whiteflies show preferences to certain Brussels sprout cultivars (i.e., antixenotic effects)?
2. Are CWF fitness parameters affected when developing on different Brussels sprout cultivars (i.e., antibiotic effects)?
3. Are there differences in the glucosinolate content among Brussels sprout cultivars, and how is this related to CWF preferences for cultivars?

## 2. Materials and Methods

### 2.1. Plant and Insect Culture

Cabbage whiteflies of the stock rearing were cultivated in wooden, gauze–covered cages (60 cm × 80 cm × 60 cm) in a greenhouse at 20–35 °C on Brussels sprout plants (cultivar Hilds Ideal), which were replaced after six weeks. From September until May, artificial light with sodium vapour lamps (Philips SON T Agro 400; Philips, Hamburg, Germany) was used to provide a long day regime of 16:8 h (L:D).

Plants for stock rearing and experiments were cultivated in Fruhstorfer Erde Typ P (Archut GmbH & Co. KG, Lauterbach, Germany) in 12 cm pots and were 5–6 weeks old when used for all indoor experiments (to reduce spread of fungal diseases). After sowing, all experimental plants were coded, so that all experiments could be performed in a blinded design. Additionally, plants for climate chamber experiments were treated once with Bayfidan (0.05% triadimenol; Cheminova Deutschland GmbH, Stade, Germany) five days before usage to avoid powdery mildew infections. To obtain synchronised individuals (no-choice experiments), two Brussels sprout plants (same cultivar as used later) were put for 48 h into the stock rearing, and afterwards all adults were removed from these plants. Then they were caged individually until adults of the next whitefly generation hatched.

We focused on commercially available and commonly used Brussels sprout cultivars (German and European market) for conventional and organic farmers and allotment holders to screen a broad range of different cultivars (see Table S1 for details). The following cultivars (kindly provided by the companies) were used: ‘Octia’, ‘AS 337’, ‘AS 336’, ‘Esperal’, ‘Speedia’, ‘Steadia’ (Agri-Saaten GmbH, Bad Essen, Germany), ‘Doric’, ‘Nautic’ (Bejo Samen GmbH, Sonsbeck, Germany), ‘Hilds Ideal’ (Hild Samen GmbH, Marbach am Neckar, Germany), ‘Brest’, ‘Breton’, ‘Bright’, ‘Brilliant’ (Nickerson–Zwaan B.V. (now Hazera Seeds B.V.), Edemissen, Germany), and ‘Content’, ‘Cyrus’, ‘Genius’ (Syngenta Agro GmbH, Maintal, Germany).

### 2.2. Screening Experiments (Antixenosis)

These experiments were performed as choice experiments in a greenhouse chamber at 22 ± 4 °C and a long day regime of 16:8 h (L:D) using sodium vapour lamps (see above) with 16 Brussels sprout cultivars, five plants per cultivar and three repetitions. The plants were arranged in a 6.4 m circle on the ground with a randomised order of cultivars and at a distance that leaves did not touch each other. After three days, 1000 randomly selected cabbage whiteflies (males and females) from the stock rearing were anaesthetised with carbon dioxide and then released in the centre of the plant circle. During the experiment, the chamber was only entered for irrigation to avoid any disturbance of the whiteflies. After seven days, all plants were individually and carefully transferred into black plastic bags (60 L), cut at the root crown and pots were discarded. The plants were frozen and later the numbers of adult whiteflies and eggs on the plant and within the bag counted using a dissecting microscope (Leica MZ 6, Leica Mikrosysteme GmbH, Wetzlar, Germany).

### 2.3. No-Choice Experiments (Antibiosis)

To evaluate cabbage whitefly fitness parameters (developmental time, larval mortality, adult weight, reproduction), experiments were done in a climate room (13.4 m^2^; Vötsch Industrietechnik GmbH, Balingen, Germany) at 21 ± 2 °C; 55% RH; 16:8 L:D; approx. 6000 Lux) and using synchronised CWF individuals (see above).

For the assessment of developmental time, mortality and adult weight, nine cultivars with five plants per cultivar were used and arranged completely randomised on climate chamber benches. Two gauze bags (20 × 30 cm) with each nine females and one male CWF were attached on the youngest fully grown leaf of each plant. Adults and bags were removed after 48 h and crawlers (L1) reduced to 20 individuals and then labelled with a fine permanent marker pen by marking the leaf surface nearby to observe the development of individuals. These larvae were checked daily for moulting and finally adult hatch. Mortality was assessed by the difference between initial egg number and finally hatched adults. After hatch, the sex of the adults and body weight was determined with 20× magnification using a dissecting microscope as above and a microbalance (Sartorius MC5; Sartorius AG, Göttingen, Germany), respectively.

In the second part of the experiment, adult females of the first experimental part were individually transferred into a clip cage on a new plant of the same cultivar. For each cultivar, five plants with three clip cages were used and egg numbers per female (fecundity) and hatching adults of the next generation (fertility) assessed weekly over the whole lifetime of the female. To overcome leaf and plant aging, all females were transferred every 14 days to a new leaf (three times during the experiment) and later onto a new plant (once during the experiment). Remaining egg clutches were covered with a clip–cage, labelled and kept until hatching of adults.

During this longer experiment, all plants were fertilised weekly with Wuxal Top N (12–4–6) 0.1% (AGLUKON Spezialdünger GmbH & Co. KG, Düsseldorf, Germany).

### 2.4. Field Trial

In 2015, a field trial was conducted on the university campus (52°23′39.4″ N, 9°42′13.3″ E, Germany) with six cultivars (Table 1) and four blocks per cultivar. Cultivars were selected according to results of the antixenosis experiments in the greenhouse, using two cultivars each, which were little, intermediate and highly preferred by CWF. Plots were arranged in a completely randomised block design in two rows (24 plots in total) and had a size of 13 m^2^ with 36 plants each (0.6 × 0.6 m distance between plants) with 0.5 m distance between plots. Planting, irrigation, fertilisation and weeding were adopted according to standard cultural practices.

In July, 11 × 100 CWF from the stock rearing were released in each intersection of four plots to avoid a patchy natural infestation of CWF. Infestation was recorded biweekly with counting egg clutches, larvae and adults on 10 plants (except plants at the edge) in every plot.

For antibiosis surveys, one female and male CWF were confined to the youngest fully grown leaf using a clip cage (Ø 3.5 cm). Per plot, two plants with five clip cages each were used. Adults were removed after 48 h and eggs counted. Thereafter, eggs, larvae, puparia, and hatched adults were counted weekly. Hatched adults were removed and their sex determined. Similar to the indoor experiments, newly hatched females (one per clip cage) were caged again and reproduction and mortality of offspring recorded until the female died, to assess lifetime fecundity parameters. Biweekly, females were caged on a new leaf of the same plant and the development of egg clutches was checked regularly until all individuals finished development, died, or the leaf fell off.

To compare contaminated plant parts (due to honeydew secretions, exuviae and growing of sooty moulds [6,15]), the degree of contamination was rated on 10 plants per plot by visually estimating button contamination on a scale from 1 (clean) to 5 (heavily contaminated).

### 2.5. Glucosinolate Analysis

Four plants from nine Brussels sprout cultivars each (except ‘Esperal’ which was not available for this experiment) were cultivated in the greenhouse and after three months transferred to the field as a potted plant. In the field, all plants were covered with a net (0.5 × 0.5 mm mesh size; Filbio PA, Hartmann–Brockhaus, Pfaffenhofen-Wagenhofen, Germany), and during the first two weeks, an additional sunscreen was applied to avoid sunburn. After two months, three leaves were cut off from two levels (upper level as first fully grown leaves and mid-level as not senescent leaves of middle part) of the plant. Both mid-vein and stem were removed from the leaves, and then transferred into a Falcon tube and finally flash frozen in liquid nitrogen. The samples were stored at −20 °C and later lyophilised using a Christ Alpha 1–4 LSC freeze drier (Christ Gefriertrocknungsanlagen GmbH, Osterode, Germany). The dry leaf material was then ground with a laboratory mill (IKA-Werke GmbH, Staufen, Germany) to a fine powder, and a sample transferred to an Eppendorf cup for glucosinolate analyses. Glucosinolate concentrations were determined as desulfo-glucosinolates using a modified method according to DIN EN ISO 9167-1, which is described in full detail in Wiesner et al. [26].

### 2.6. Statistics

All statistical analyses were performed using R Version 3.2.2 [27].

Analyses of the choice experiments (log transformed) in the greenhouse were done with a deviance analysis using a generalised linear model (GLM) based on the log-link function and assumption of a quasi-Poisson distribution to correct for over-dispersion.

In no-choice experiments (climate chamber), statistical analyses on developmental times (log transformed), adult weight and reproduction (log+1 transformed) were performed with linear mixed models (LMM, using the lmer function in R package lme4; [28]) including plant cultivars as a fixed effect and single plants as a random effect. This was followed by an ANOVA (with Kenward–Roger’s approximation of denominator degrees of freedom). As significant differences were found in cultivars (*p* = 0.0018) and sex (*p* < 0.00001) with respect to adult weight, they were separately analysed using a linear model (function lm). All pairwise comparisons were then done using an analogue of Tukey’s test (R package lsmeans; [29]).

Mortality was analysed using a generalised linear mixed model (GLMM using the glmer function in R package lme4) assuming a binominal data distribution. The model included plant cultivars as a fixed effect and single plants and an additional variance effect between individuals as random effects (see above). Pairwise comparisons were then done as above using lsmeans.

For infestation and plant attractiveness in the field, data were log+1 transformed and analysed using a linear mixed model (LMM/lmer) function separately for each count date. In the model, variance between plots and residual variance between single plants were included as random effects and cultivar means and block differences as fixed effects. Again, pairwise comparisons were done for cultivar differences using an analogue of Tukey’s test. Mortality and reproduction were analysed as above, using an LMM or GLMM.

To analyse the differences in glucosinolate concentrations (concentrations of all substances combined) of a selected number of cultivars, we applied a MANOVA using the manova function of R and Wilks’ λ-test as an overall test. Between cultivars, differences of individual glucosinolates, total glucosinolate and glucosinolate subgroups were modelled using a GLM using a Gaussian data distribution and pairwise comparisons as above (lsmeans package).

Contamination levels were compared with a likelihood ratio test of two cumulative link mixed models (CLMM). The models included average cultivar differences or a wildcard as a fixed effect and the variance between plots as random effects. Finally, pairwise comparisons were performed with lsmeans by comparing the average tendency to higher rating levels.

## 3. Results

### 3.1. Screening Experiments (Antixenosis)

**Figure 1:**
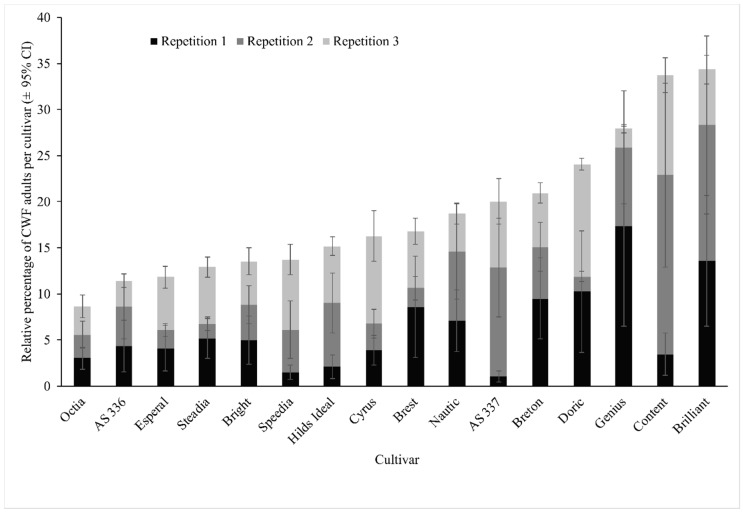
Infestation of 16 Brussels sprout cultivars by adult *A. proletella* (Hemiptera: Aleyrodidae) in three screening experiments with circular design in a greenhouse. Each stacked bar represents the relative percentage of *A. proletella* counts (±95% confidence interval) per cultivar of each of the three separate experiments. In all experiments, 1000 cabbage whiteflies (CWFs) were released, and after seven days, each plant was evaluated for infestation. The sum of all recaptured individuals per experiment is 100 per cent.

Figure 1 shows the mean number of adult cabbage whiteflies per cultivar of the three cultivar screenings which were performed as choice experiments. Although no significant differences were found (GLM, F_(15,220)_ = 1.25, *p* = 0.2358), a relatively obvious sequence of less (left side) and more infested (right side) cultivars is visible, suggesting differences in cultivar attractiveness, though among repetitions, results for certain cultivars are not always consistent. The explanatory power of the experiment is reduced due to the high variation indicated by the confidence intervals (Figure 1), resulting from a very heterogeneous infestation of plants in the circles (i.e., some plants were not, others highly infested). The number of eggs (Figure S1) had a similar distribution compared to adults with a similar sequence of cultivars having a high number of eggs or not; further, the differences were not significant (GLM, F_(15,220)_ = 7.03, *p* = 0.7817). However, we left out egg numbers from further interpretation, as the adults were not synchronised for this (i.e., a mix of different sexes and ages). From these results, cultivars were selected for antibiosis experiments in the greenhouse and the field trial to have a feasible number of cultivars with different resistance potential.

The average recapture rate of CWF in these experiments was 78% (66.3%–98.3%).

### 3.2. No-Choice Experiments (Antibiosis)

Table 1 displays summarised fitness parameters of the no-choice experiments in a climate chamber to evaluate the suitability of selected cultivars for cabbage whitefly development. The results indicate that not all fitness parameters were affected by cultivar differences as three of them show no significant differences: Life-time fertility (F_(8,97)_ = 0.9034, *p* = 0.5170), fecundity rate (F_(8,97)_ = 0.4998; *p* = 0.8459) and female longevity (F_(8,97)_ = 0.519; *p* = 0.8396). All others show significant differences (Table 1), suggesting a significant effect of cultivars on these parameters (developmental time: F = 9.0972, *p* < 0.0001; female weight: F = 4.33, *p* = 0.0018; mortality: GLMM *p* < 0.05). There is no clear trend that those cultivars, which were estimated as attractive in the cultivar screening (choice experiments), were also more suitable for CWF development and vice versa. For example, CWF on the attractive cultivar Content showed similar developmental time and mortality compared to the less attractive cultivars Octia or Bright, whereas among the less attractive cultivars Octia and Esperal significant differences in developmental time were found. However, highest weights were found in ‘Hilds Ideal’ and ‘Content’ (an attractive cultivar) and lowest in ‘Doric’ and ‘Octia’ (a less attractive cultivar) which matches with cultivar attractiveness. Overall mortality was lowest on the cultivar Hilds Ideal, which was also used for stock rearing of CWF, indicating a possible effect of customisation to this cultivar. Female CWF had on average significantly more weight compared to males independent of the cultivar (mean of males = 0.045 ± 0.009 mg; mean of females = 0.081 ± 0.016 mg; LMM, *p* < 0.001).

### 3.3. Field Trial

**Figure 2:**
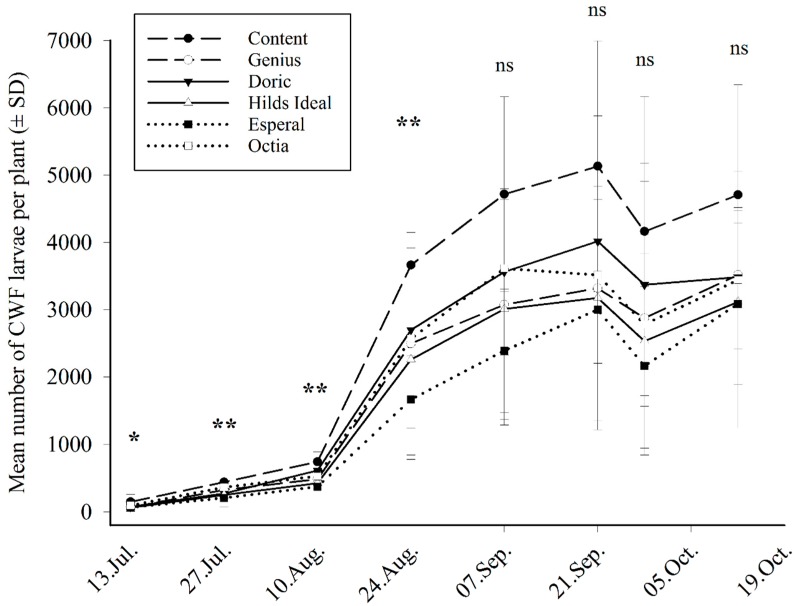
Development of *A. proletella* (average counts of larvae per plant) on different Brussel sprout cultivars in a field trial (±SD). Dashed lines show attractive, solid lines intermediate and dotted ones unattractive cultivars, according to previous experiments in a greenhouse. Stars indicate significant differences between cultivars at a particular date (LMM, *p* < 0.05); significance codes: ** = 0.01 * = 0.05.

The field trial included a selection of six cultivars which were evaluated in greenhouse experiments (Figure 1) as less (‘Octia’, ‘Esperal’), intermediate (‘Hilds Ideal’, ‘Doric’) and highly (‘Genius’, ‘Content’) attractive to CWF. In the field, the infestation with CWF larvae (Figure 2) was partly similar to the greenhouse experiments, with ‘Content’ having the highest and ‘Esperal’ having the lowest infestation. The differences are significant from the first counting date (13 July 2015) until 24 August 2015 for larvae (LMM, *p* < 0.05). Later differences show a similar tendency, but differences were not significant because of the increasing standard deviations (Figure 2). Adults and egg clutches showed a similar pattern of increase with time, although egg clutches showed no significant differences (LMM, *p* > 0.05 for all dates), and adults showed significant differences on three dates (LMM, 28 July 2015: F = 6.62, *p* = 0.0019; 25 August 2015: F = 11.69, *p* < 0.0001; 8 September 2015: F = 6.14, *p* = 0.00275). Surprisingly, cultivar ‘Genius’ had only an intermediate infestation with larvae, which was not significantly different from ‘Octia’. With regard to antibiosis parameters, fecundity rates of CWF did not differ between all cultivars (LMM, F = 1.76, *p* = 0.1866), and in offspring mortality, only offspring developing on cultivars ‘Esperal’ and ‘Hilds Ideal’ had a significantly higher mortality than offspring on ‘Content’ (GLMM, *p* < 0.05). The degree of contamination showed significant differences (CLMM, *p* < 0.0001) with ‘Esperal’ as significantly least and ‘Genius’ as most contaminated cultivar (Table 2). There was no correlation between degree of contamination and infestation with adults or larvae (Spearman’s rank-correlation coefficient r_s_ = 0.0105, *p* = 0.8710 for adults; r_s_ = 0.1630, *p* = 0.0116 for larvae).

### 3.4. Glucosinolates

Three alkenyl (2-propenyl, 3-butenyl, (R)-2-hydroxy-3-butenyl), two methylsulfinyl (3-methylsulfinylpropyl, 4-methylsulfinylbutyl) and four indole (Indolyl-3-methyl, 4-hydroxy-3-indolylmethyl, 4-methoxy-3-indolylmethyl, 1-methoxy-3-indolylmethyl) glucosinolates were found in the Brussels sprout leaves with varying concentrations (Table 3, see also Table S2 for results of all cultivars and individual glucosinolates). In addition, significant differences of several glucosinolates between cultivars were detected at both leaf levels (GLM, *p* < 0.05; Table S2), for the main glucosinolate subgroups (GLM, *p* < 0.05, except indole glucosinolates) and for total concentrations at mid-level leaves (GLM, *p* = 0.0051; Table 3). A one-way MANOVA revealed a significant multivariate main effect for cultivar (Wilks’ λ = 0.000198, approx. F_(8,89)_ = 21.292). Given the significance of the overall test, the univariate main effects were examined by single ANOVAs. Significant univariate main effects were obtained for all glucosinolates except (R)-2-hydroxy-3-butenyl (*p* ≤ 0.0013). ‘Doric’ and ‘Genius’ had the highest total glucosinolate concentrations in the upper leaves, and ‘Genius’ and ‘Hilds Ideal’ in the mid leaves, although no significant differences were found (GLM, *p* > 0.05). A trend to higher concentrations of indole, alkenyl and methylsulfinyl glucosinolates could be found especially in mid-level leaves (Table 3), with ‘Octia’ having lowest concentrations and ‘Genius’, ‘Doric’ and ‘Hilds Ideal’ having highest concentrations. ‘Esperal’ was tested in a different experiment later, and low glucosinolate concentrations were found (data not shown). These data are excluded from Table 3, as experimental conditions were different. Finally, in all cultivars, upper leaves had higher total glucosinolate concentrations (GLM, *p* < 0.01) compared to mid-level leaves.

## 4. Discussion

Our results indicate that some commercialised Brussels sprout cultivars show at least a partial resistance against cabbage whitefly infestations. Differences in the infestation of cultivars indicate presence of antibiosis mechanisms and differences in mortality and developmental time presence of antixenosis mechanisms. Resistance of Brassicas against CWF was investigated in several studies with positive results [6,15,21,22,23,30,31,32], but most of the studies compared the host suitability among different *Brassica* groups, such as cauliflower, kale or white cabbage. Extensive studies of cultivars within one group, such as Brussels sprout, are rare. Antixenosis of Brussels sprout cultivars has been documented previously only by Hirthe and Jakobs [20], who found in field trials that the cultivars ‘Esperal’, ‘Genius’ and ‘Steadia’ were less infested, and ‘Doric’ was the most infested cultivar. These results are partly inconsistent with ours, and possible reasons might be that plants in field trials (being per se more exposed to confounding factors of the environment) are affected by additional biotic and abiotic stresses (e.g., pests, diseases, heat, radiation, etc.) and different cultivation, such as fertiliser use with impact on glucosinolate and nitrogen levels [33] or irrigation regimes. Both might have affected the suitability of host plants and preference of herbivores [32]. Other cultivar studies were performed by Muñiz and Nebreda [19], Nebreda et al. [22] and Broekgaarden et al. [23], who found differences in CWF infestations and host suitability of commercialised cauliflower, broccoli and white cabbage cultivars, indicating antixenosis and antibiosis mechanisms, respectively. In detail, resistance against CWF was studied solely for white cabbage (*B. oleracea* var. *capitata* f. *alba*), and a considerably resistant cultivar was identified. Their results indicate that the detected resistance is antibiosis based, phloem specific and dependent on plant age [17,23]. In contrast to other studies, they could not find antixenosis mechanisms, which might be caused by the low number of cultivars surveyed, but also because white cabbage seems to be a relatively poor host plant for CWF [6].

Surprisingly, in several of the studied cultivars, antibiosis and antixenosis mechanisms were not found in the same cultivar, which would be reasonable in terms of herbivore fitness (preference–performance hypothesis; [34]). Moreover, a careful host plant selection seems appropriate, as the sessile larvae have to stay a long time on the same plant without the possibility to switch. Apart from the fact that some choice results of certain cultivars among the three experimental repetitions were inconsistent, and the choices did not significantly differ (and hence the attractiveness of these cultivars is debatable), resistance based on antibiosis and antixenosis mechanisms is not necessarily found in the same cultivar (e.g., [23,35]) and do not dependent on each other. It is not always the case that females select host plants favourable for their offspring [36,37]; instead, they may select high-quality host plants for feeding and thus boost the number of eggs laid. Additionally, there can be a trade-off between costs of dispersal and host plant choice [38]. In contrast, the found coincidence of antibiotic and antixenotic effects in some of our cultivars (e.g., in ‘Octia’ and ‘Genius’) could indicate that the CWF is able to assess plant quality very early without probing the plant. Either way, we found a comparable trend in the development of adult, larva, and egg clutch numbers in all cultivars, suggesting that CWF oviposition preference was not influenced by host quality, since even on relatively poor hosts, high numbers of offspring developed (but see below, spill-over effect). In the context of host selection, there are many unknowns for CWF, such as the importance of plant odours vs. colours, the effect of plant shape and the role of plant compounds such as glucosinolates (see below) or flavonoids. Few studies [23,30] have addressed some of these topics, and results suggest that both colour and odour are used as cues for host plant selection, although the preferred colours and plant volatiles are unknown except for a general preference for yellow as in most hemipterans (e.g., [39]).

Both antixenosis and antibiosis could be observed in our climate chamber, greenhouse and field trials, suggesting that the conditions of climate chambers with artificial light and greenhouses with reduced UV did not confound the outcomes, although an effect (i.e., a reduced resistance) is likely. Especially UV can trigger and add to resistance mechanisms of Brassicas, respectively [40]. Therefore, we rather underestimated the resistance potential of the studied Brussels sprout cultivars in the indoor experiments, and, under less artificial conditions, effects could have been more pronounced. In our field trial, only a moderate resistance (both antibiosis and antixenosis) could be found as well. In field trials, plants are affected by additional stresses as mentioned above. Even an “induced susceptibility” of host plants was reported, exemplified by *Bemisia tabaci* (Genn.) (Hemiptera: Aleyrodidae) on tomato, which resulted in aggregation of this pest to benefit from the metabolic changes induced by conspecifics [41]. This phenomenon might also occur in CWF infestations in the field (CWF also aggregates), but not in experiments using clip cages with isolated individuals. Therefore, isolated individuals might experience tougher conditions, which are intensified by possible impacts of clip cages on leaf metabolism [42]. Finally, when high pest population densities develop, they can spill over to other plants and blur the effects (“associational susceptibility”; [43,44]). Thus, it remains partly unclear if the resistance of the studied commercialised Brussels sprout cultivars is actually only moderate or if experimental conditions are in part the reason for the stated moderate resistance.

Glucosinolates are considered as plant defence compounds against herbivores and pathogens (bottom-up regulation) which include both constitutive and induced chemical defence [25]. In contrast to aphids, cabbage whiteflies cannot avoid glucosinolate-enriched plant parts through behavioural responses (i.e., switching to leaves with low glucosinolate content) because adults have to oviposit on younger leaves, which possess a higher glucosinolate content [25] (own data). The larvae, being sessile after the first instar, then have to feed on the leaves for a relatively long time in order to complete their development before the leaf falls off. However, if possible, females could avoid plants with higher glucosinolate content, but according to our results, they do not do so. There was no preference for cultivars with lower glucosinolate content but rather the opposite, since cultivars such as ‘Content’ and ‘Genius’ showed a comparatively enhanced total glucosinolate content (especially in the mid-level leaves) and belong to the preferred cultivars. The same trend was found in the field trial of Hirthe and Jacobs [20]. In addition, antibiotic effects are not evident in glucosinolate-rich cultivars. This indicates that CWF can cope with such glucosinolate concentrations and seems to have evolved appropriate mechanisms for this (see [25]) which are unidentified yet. For the generalist whitefly *B. tabaci*, it has been reported that several detoxification enzymes are present or induced when feeding on plants containing glucosinolates, demonstrating a possible detoxification mechanism in whiteflies [45]. However, the results of the glucosinolate analyses and cultivar preference are partly inconsistent because in the preferred cultivars both high and low glucosinolate level cultivars (in respect to both total and subgroup content) were present. This was also observable for individual glucosinolates, such as 2-propenyl (sinigrin) and (R)-2-hydroxy-3-butenyl (progoitrin). The study by Santolamazza-Carbone et al. [44] also revealed that different glucosinolate levels of sinigrin, 3-methylsulfinylpropyl (glucoiberin) and indolyl-3-methyl (glucobrassicin) did not affect CWF abundance on kale. In contrast, these results are inconsistent with the study by Newton et al. [46], who found that the infestation of wild cabbage populations by *A. proletella* was decreasing with higher proportions of plants producing sinigrin, whereas progoitrin had no effect. An explanation might be that wild cabbages could have distinct higher glucosinolate contents [47,48], and CWF then try to avoid such glucosinolate-rich plants. Breeding of Brussels sprout cultivars nowadays aims at reducing glucosinolate contents to achieve a milder taste [49,50], and breeders even advertise this trait in their cultivar descriptions. Therefore, several reasons for the conflicting findings are possible: Glucosinolates are not important as a cue for host plant selection and fitness of CWF and thus as a plant resistance factor against CWF [51]; however, this seems unlikely with regards to the toxicity and costs to cope with glucosinolates and their breakdown metabolites. It appears more important that phloem feeders such as CWF induce just limited damage in the plant tissue and hence avoid damaging cells containing glucosinolates and/or myrosinase enzymes (glucosinolates are mostly in found in vacuoles but also in phloem sap; [52,53]). Strong plant reactions affecting glucosinolate content are reported upon aphid feeding (e.g., [54]), showing that this picture is surely incomplete. For some glucosinolates, such as alkenyl glucosinolates, it is suggested that they play an important role in the response against microbial pathogens [55], so that our results might also be biased by microbial infections of the field trial plants, which we did not notice. Finally, results of glucosinolate analyses are just “snap shots”, which can vary depending on many biotic and abiotic parameters (see above) and which can change rapidly [56,57]. When a CWF infestation starts, glucosinolate contents of the plants are changing, which obviously alters the conditions for new colonisers and subsequent generations. Therefore, the explanatory power of such non-recurring analyses on a continuous process of plant colonisation and pest development might be low. Consequently, in future, such analyses should be performed as time course experiments to better understand the relationship of infestations and changing glucosinolate levels.

The main damages caused by heavy CWF infestations are contaminated leaves and buttons due to honeydew secretions and growing of sooty moulds (e.g., [6,15]). Firstly, leaves are covered, which impairs photosynthesis resulting in reduced yields [1,2], and secondly, contamination level is decisive for whether the harvest (i.e., buttons) is marketable or not. Although buttons can be cleaned to some extent, too contaminated ones are rejected by the market due to quality standards such as by UNECE [58]. Our data show significant differences in the degree of contamination (except in ‘Content’) but no correlation between degree of contamination and infestation. As possible reasons, plant architecture with leaf arrangement and their inclination angle are considered, as a certain arrangement of leaves seems to cover buttons better than others and thus prevents contamination [20]. The effects of such plant characteristics are still not well understood, and further research is necessary, as this might be an alternative approach to manage CWF infestations.

## 5. Conclusions

The present study shows that some commercialised Brussels sprout cultivars show a moderate resistance against cabbage whiteflies based on antibiosis and antixenosis mechanisms. The principles behind the found resistance patterns are so far unknown, as glucosinolates seem not to be decisive for host selection and detected antibiotic effects. For plant protection strategies, this resistance is certainly not strong and consistent enough as a stand-alone measure to deal with high levels of pest pressure, since in most cultivars estimated as resistant, only few antixenotic effects could be observed, and in the field, all cultivars were finally highly infested. However, such less susceptible cultivars can become a component of multi-layered integrated pest management strategies, since this does not interfere with most approaches and can then even be economically advantageous [14]. Moreover, for all integrated and biological plant protection strategies, multi-level approaches are a standard, and cultivars that are more resistant could form the base for further improvements [59]. The availability of suitable Brussels sprout cultivars seems to be more problematic because present-day cultivars are usually bred for traits such as yield, nutritional quality, or machine processing and not for traits suitable for organic production (e.g., pest resistance, increased competitiveness against weeds, increased nutrient-use efficiency; [60,61]). In addition, many recent cultivars are CMS (cytoplasmic male sterility) hybrids—A technique that is incompatible with organic production standards such as [62].

## Figures and Tables

**Table 1:** Results of antibiosis experiments, including developmental time (egg to adult), mortality (combined egg and larval), fecundity rate (number of eggs per day and female), life-time fertility (number of offspring per female over whole lifetime), female weight (hatched adults), and longevity of females (means ± SD) of cabbage whiteflies (*A. proletella*) after development on different Brussels sprout cultivars in a climate chamber at 21 ± 2 °C, 55% rH, 16:8 L:D. Within a column, means with different letters are significantly different (generalised linear mixed model (GLMM)/LMM, *p* ≤ 0.05).

Cultivar	Total Developmental Time (Days)	Mortality (%)	Fecundity Rate	Life-Time Fertility	Female Weight (mg)	Longevity (Days)
Octia	28.0 ± 3.35 ac	27 ± 10 ac	3.1 ± 1.3 a	153.5 ± 88.4 a	0.074 ± 0.018 ab	63 ± 24 a
Esperal	25.0 ± 2.98 b	25 ± 8.0 ac	3.5 ± 0.5 a	134.8 ± 71.5 a	0.085 ± 0.015 cd	53 ± 30 a
Bright	26.6 ± 2.49 ab	41 ± 20 ab	3.6 ± 1.7 a	95.3 ± 52.4 a	0.078 ± 0.019 abc	48 ± 18 a
Speedia	28.0 ± 2.95 ac	39 ± 13 ab	3.0 ± 0.8 a	138.2 ± 118.5 a	0.079 ± 0.013 abc	64 ± 43 a
Hilds Ideal	27.7 ± 2.70 ac	19 ± 7.0 c	3.3 ± 0.7 a	116.6 ± 53.6 a	0.091 ± 0.012 d	49 ± 29 a
Doric	27.6 ± 2.69 ac	33 ± 15 abc	2.5 ± 0.9 a	91.4 ± 60.2 a	0.071 ± 0.016 a	53 ± 26 a
Genius	26.6 ± 2.06 a	44 ± 20 b	3.1 ± 1.5 a	93.8 ± 85.4 a	0.080 ± 0.014 bc	47 ± 24 a
Brilliant	28.5 ± 4.08 c	27 ± 14 ac	2.8 ± 0.7 a	117.3 ± 59.9 a	0.075 ± 0.015 ab	56 ± 24 a
Content	26.7 ± 1.95 a	37 ± 12 ab	3.3 ± 0.9 a	123.8 ± 62.6 a	0.091 ± 0.012 d	57 ± 23 a
Grand mean	27.01 ± 2.82	32.0 ± 15.6	3.08 ± 1.06	115.8 ± 70.3	0.081 ± 0.015	53.8 ± 25.6

**Table 2:** Cabbage whitefly antibiosis results (offspring mortality, fecundity (number of eggs per day and female) and estimated degree of contamination caused by CWF infestation of the field trial (means ± SD). Within a column, means with different letters are significantly different (GLMM/LMM, *p* ≤ 0.05).

Cultivar	Mortality (%)	Fecundity Rate	Degree of Contamination
Octia	79 ± 27 ab	2.8 ± 1.4 a	3.3 ± 0.6 a
Esperal	89 ± 20 b	3.2 ± 1.5 a	1.4 ± 0.5 c
Hilds Ideal	88 ± 20 b	2.9 ± 1.4 a	3.3 ± 0.6 a
Doric	80 ± 22 ab	3.4 ± 1.6 a	4.3 ± 0.5 b
Genius	92 ± 12 ab	1.6 ± 1.1 a	2.3 ± 0.7 d
Content	68 ± 29 a	3.4 ± 1.6 a	3.4 ± 0.9 a
Grand mean	81.7 ± 24	3.03 ± 1.5	2.95 ± 1.1

**Table 3:** Mean concentrations of total glucosinolates and main glucosinolate subgroups (indole, alkenyl and methylsulfinyl glucosinolates (GS)) of five Brussels sprout cultivars at two leaf levels (±SD). Within a column, means of same leaf level with different letters are significantly different (generalised linear model (GLM), *p* ≤ 0.05).

	GS Concentration (µmol/g Dry Mass)							
	Upper Level Leaves				Mid–Level Leaves			
Cultivar	Total GS	Indole GS	Alkenyl GS	Methylsulfinyl GS	Total GS	Indole GS	Alkenyl GS	Methylsulfinyl GS
Octia	55.90 ± 32.40 a	29.99 ± 18.21 a	10.35 ± 7.94 b	15.56 ± 8.10 ab	11.60 ± 20.18 b	7.06 ± 12.54 a	1.17 ± 1.84 b	3.37 ± 5.82 c
Hilds Ideal	60.46 ± 11.19 a	30.07 ± 6.94 a	11.99 ± 5.44 b	18.40 ± 5.71 a	37.45 ± 14.93 a	15.38 ± 8.47 a	6.06 ± 4.78 ab	16.01 ± 8.14 a
Doric	65.11 ± 25.85 a	35.11 ± 19.85 a	18.81 ± 5.01 a	11.19 ± 2.72 b	22.68 ± 13.91 ab	9.47 ± 6.08 a	6.89 ± 4.86 a	6.32 ± 4.66 bc
Genius	62.88 ± 19.41 a	35.97 ± 18.55 a	11.67 ± 5.36 b	15.24 ± 2.80 ab	32.79 ± 23.46 a	14.80 ± 12.40 a	6.54 ± 5.38 a	11.46 ± 6.46 ab
Content	61.57 ± 19.65 a	32.74 ± 14.22 a	10.63 ± 4.08 b	18.19 ± 3.20 a	29.19 ± 16.10 ab	12.32 ± 7.49 a	6.12 ± 4.92 ab	10.75 ± 4.63 ab

## Supplementary Materials

The following are available online at https://www.mdpi.com/2075-4450/11/1/56/s1, Figure S1: Mean egg numbers in screening experiments for antixenosis, Table S1: List of cultivars used and companies for the different experiments and analyses, Table S2: Concentrations of glucosinolates of nine Brussels sprout cultivars at two leaf levels.
