# Supplementary material for: Importance of Antixenosis and Antibiosis Resistance to the Cabbage Whitefly (Aleyrodes proletella) in Brussels Sprout Cultivars
Source: Insects. 2020 Jan 17;11(1):56. doi: 10.3390/insects11010056 (PMC7022423; doi:10.3390/insects11010056)
Supplement: Supplementary file 1 [file insects-11-00056-s001.pdf]

Table. S1: List of cultivars used and companies for the different experiments and analyses.

Except Hilds Ideal and AS 336, 337 all cultivars are F1 hybrids. Of the latter two, status is unknown.

Antixenosis (greenhouse)	Antibiosis (climate chamber)	Field trial (both resistance)	Glucosinolate analyses	CMS Status
Octia (Agri-Saaten)	Octia	Octia	Octia	without
AS 336 (Agri-Saaten)*				?
AS 337 (Agri-Saaten)*				?
Esperal (Agri-Saaten)	Esperal	Esperal	Esperal	without
Speedia (Agri-Saaten)	Speedia			without
Steadia (Agri-Saaten)			Steadia	without
Doric (Bejo Samen)	Doric	Doric	Doric	without
Nautic (Bejo Samen)				without
Hilds Ideal (Hilds Samen)	Hilds Ideal	Hilds Ideal	Hilds Ideal	without
Brest (Nickerson-Zwaan)			Brest	with
Breton (Nickerson-Zwaan)				with
Bright (Nickerson-Zwaan)	Bright		Bright	with
Brilliant (Nickerson-Zwaan)	Brilliant			?**
Content (Nickerson-Zwaan)	Content	Content	Content	?**
Cyrus (Syngenta Agro)			Cyrus	without
Genius (Syngenta Agro)	Genius	Genius	Genius	without

*: Experimental cultivars supplied by the company, which are not on the market

**: presumably without CMS because old cultivars

Table S2: Concentrations of glucosinolates (µmol/g dm ± SD) of nine Brussels sprout cultivars at two leaf levels. GLM Tukey-Test, $p < 0.05$; letters are only comparable between columns (cultivars) within a row.

	Brussels sprout cultivar and GLS concentration (µmol/g dm ± SD) in **upper leaf level**								
Glucosinolates	Content	Hilds Ideal	Bright	Cyrus	Doric	Brest	Genius	Octia	Steadia
3-methylsulfinyl-propyl	8.95 ± 1.57 (cd)	5.40 ± 1.07 (e)	9.79 ± 2.33 (bc)	10.6 ± 1.80 (bc)	5.68 ± 1.36 (de)	8.29 ± 3.13 (ce)	12.9 ± 2.33 (ab)	10.5 ± 5.47 (bc)	16.1 ± 1.65 (a)
(R)-2-hydroxy-3-butenyl	3.78 ± 1.42 (a)	4.55 ± 3.14 (a)	6.50 ± 3.69 (a)	3.06 ± 1.49 (a)	6.27 ± 2.70 (a)	4.82 ± 4.16 (a)	3.00 ± 1.63 (a)	3.95 ± 3.08 (a)	4.79 ± 3.67 (a)
2-propenyl	5.16 ± 2.05 (c)	6.03 ± 1.92 (bc)	11.4 ± 3.97 (b)	9.65 ± 3.08 (bc)	8.18 ± 2.25 (bc)	21.5 ± 6.74 (a)	7.64 ± 3.53 (bc)	5.88 ± 4.50 (c)	7.54 ± 3.60 (bc)
4-methylsulfinyl-butyl	9.24 ± 1.65 (b)	13.0 ± 5.04 (a)	5.38 ± 1.65 (cdf)	1.27 ± 0.44 (ef)	5.51 ± 1.52 (cd)	1.41 ± 0.99 (e)	2.33 ± 0.87 (de)	5.11 ± 2.73 (cdf)	8.04 ± 4.05 (bc)
3-butenyl	1.70 ± 0.67 (cd)	1.41 ± 1.01 (cd)	6.79 ± 2.38 (a)	1.09 ± 0.57 (cd)	4.36 ± 0.67 (b)	1.99 ± 1.54 (c)	1.03 ± 0.58 (cd)	0.53 ± 0.43 (d)	1.40 ± 0.72 (cd)
4-hydroxy-3-indolylmethyl	0.21 ± 0.12 (b)	0.13 ± 0.05 (bc)	0.17 ± 0.13 (bc)	0.15 ± 0.06 (bc)	0.09 ± 0.05 (c)	0.14 ± 0.07 (bc)	0.05 ± 0.02 (c)	0.08 ± 0.06 (c)	0.47 ± 0.15 (a)
Indolyl-3-methyl	31.7 ± 13.90 (a)	29.5 ± 6.77 (a)	6.03 ± 4.05 (b)	21.9 ± 0.06 (ab)	33.9 ± 19.88 (a)	23.0 ± 11.7 (ab)	34.7 ± 18.39 (a)	28.7 ± 17.70 (a)	39.8 ± 22.8 (a)

4-methoxy-3-indolylmethyl	0.64 ± 0.21 (c)	0.34 ± 0.19 (e)	0.63 ± 0.25 (de)	0.89 ± 0.29 (ade)	1.04 ± 0.51 (acd)	0.86 ± 0.35 (bcd)	1.22 ± 0.32 (ab)	1.18 ± 0.53 (ad)	1.47 ± 0.56 (a)
1-methoxy-3-indolylmethyl	0.16 ± 0.11 (a)	0.14 ± 0.12 (ab)	0.16 ± 0.19 (a)	0.03 ± 0.02 (bc)	0.06 ± 0.06 (ac)	0.01 ± 0.01 (c)	0.03 ± 0.02 (bc)	0.02 ± 0.01 (c)	0.01 ± 0.02 (c)

	Brussels sprout cultivar and GLS concentration (µmol/g dm ± SD) in **mid leaf level**								
Glucosinolates	Content	Hilds Ideal	Bright	Cyrus	Doric	Brest	Genius	Octia	Steadia
3-methylsulfinyl-propyl	5.52 ± 2.29 (bd)	4.80 ± 1.71 (bd)	4.10 ± 2.63 (bd)	8.71 ± 5.61 (abc)	2.97 ± 1.93 (d)	3.53 ± 4.09 (cd)	10.1 ± 5.53 (a)	2.55 ± 3.91 (d)	8.12 ± 3.55 (ab)
(R)-2-hydroxy-3-butenyl	2.03 ± 2.33 (a)	1.66 ± 2.48 (ab)	0.45 ± 0.40 (ab)	0.87 ± 0.93 (ab)	1.49 ± 1.22 (ab)	0.20 ± 0.24 (b)	1.08 ± 1.43 (ab)	0.24 ± 0.78 (b)	0.33 ± 0.46 (ab)
2-propenyl	3.25 ± 1.92 (ac)	3.60 ± 2.14 (ac)	3.13 ± 1.63 (ac)	6.10 ± 2.65 (a)	3.72 ± 2.48 (ac)	4.58 ± 4.83 (ab)	4.94 ± 3.49 (ab)	0.89 ± 0.99 (c)	1.71 ± 1.39 (bc)
4-methylsulfinyl-butyl	5.23 ± 2.37 (b)	11.2 ± 6.61 (a)	2.00 ± 1.27 (bc)	0.83 ± 0.82 (c)	3.35 ± 2.80 (bc)	0.32 ± 0.37 (c)	1.33 ± 0.97 (c)	0.82 ± 1.96 (c)	2.08 ± 1.56 (bc)
3-butenyl	0.85 ± 0.72 (bc)	0.80 ± 0.65 (bc)	1.10 ± 0.74 (ab)	0.43 ± 0.20 (bc)	1.68 ± 1.26 (a)	0.27 ± 0.34 (c)	0.52 ± 0.53 (bc)	0.04 ± 0.11 (c)	0.19 ± 0.25 (c)

4-hydroxy-3-indolylmethyl	0.13 ± 0.05 (bc)	0.11 ± 0.06 (bc)	0.07 ± 0.05 (bc)	0.19 ± 0.12 (bc)	0.08 ±0.11 (bc)	0.19 ± 0.15 (b)	0.10 ± 0.06 (bc)	0.03 ± 0.06 (c)	0.46 ± 0.23 (a)
Indolyl-3-methyl	11.6 ± 7.51 (ab)	14.9 ± 8.50 (a)	0.73 ± 0.53 (c)	6.65 ± 6.46 (ac)	8.34 ± 6.48 (ac)	2.89 ± 3.55 (bc)	13.8 ± 12.44 (a)	6.15 ± 12.4 (ac)	13.0 ± 6.81 (ab)
4-methoxy-3-indolylmethyl	0.53 ± 0.33 (bc)	0.28 ± 0.12 (c)	0.55 ± 0.24 (bc)	0.55 ± 0.19 (ac)	1.04 ± 0.72 (a)	0.72 ± 0.19 (ac)	0.92 ± 0.30 (ab)	0.87 ± 0.39 (ab)	0.66 ± 0.45 (ac)
1-methoxy-3-indolylmethyl	0.08 ± 0.08 (a)	0.05 ± 0.05 (ab)	0.08 ± 0.11 (a)	0.05 ± 0.04 (ab)	0.02 ± 0.02 (ab)	0.02 ± 0.02 (ab)	0.00 ± 0.00 (b)	0.01 ± 0.02 (b)	0.00 ± 0.00 (b)
